# Correction to “Cross-Link Density and Strain-Induced Molecular Chain Orientation Relationship in Hydrogenated Nitrile Butadiene Rubbers”

**DOI:** 10.1021/acs.macromol.6c01700

**Published:** 2026-07-01

**Authors:** Giuseppe Femina, Odda Ruiz de Ballesteros, Raffaele Marzocchi, Martin van Duin, Christoph Gögelein, Paul Sotta, Finizia Auriemma

In the original publication, the data shown in Figure 4B are not presented in a fully correct way and this may be misleading to the reader. This point was drawn to our attention following a personal communication with Prof. Kay Saalwächter. We wish to clarify the definition of the average *D*_res_ value reported in the original manuscript.

The data refer to the normalized DQ signal *I*_*nDQ*_(*t*), which is expressed as a Freeholm integral of the form (see Equation S17 in the Supporting Information file):

1 $$I_{nDQ}\left(t\right)=\int_{D_{\min}}^{D_{\max}}P\left({\mathrm{D̃}}_{res}\right)\;\mathrm{Kern}\left(t,{\mathrm{D̃}}_{res}\right)\;d{\mathrm{D̃}}_{res}$$

where $\mathrm{Kern}\left(t,{\mathrm{D̃}}_{res}\right)$ is the Kernel function, corresponding to a hypothetically fully homogeneous sample, which was chosen according to ref 1.

The normalized distribution $P\left({\mathrm{D̃}}_{res}\right)$ was assumed to be log-normal (see Equation S19 in the Supporting Information):

2 $$P\left({\mathrm{D̃}}_{res}\right)=\frac{1}{{\mathrm{D̃}}_{res}\sigma \sqrt{2\pi }}\;\exp\left[-\frac{{\left(\ln\left({\mathrm{D̃}}_{res}\right)-\mu \right)}^{2}}{2\sigma^{2}}\right]$$

It follows that eq 1 can be rewritten:

3 $$I_{nDQ}\left(t\right)=\int_{{ũ}_{\min}}^{{ũ}_{\max}}P\left(ũ\right)\;\mathrm{Kern}\left(t,{\mathrm{D̃}}_{res}\left(ũ\right)\right)\;dũ$$

where the variable $ũ\equiv \ln\left({\mathrm{D̃}}_{res}\right)$ and P(ũ) is the Gaussian distribution:

4 $$P\left(ũ\right)=\frac{1}{\sigma \sqrt{2\pi }}\;\exp\left[-\frac{{\left(ũ-\mu \right)}^{2}}{2\sigma^{2}}\right]$$

The relevance of using a log-normal ${\mathrm{D̃}}_{res}$ distribution $P\left({\mathrm{D̃}}_{res}\right)$ was first outlined by Lorthioir 2013 ^2^ and discussed in detail in ref 3.

The width σ of the distribution is a dimensionless quantity. It follows from the Gaussian shape of the $P\left(\ln\left({\mathrm{D̃}}_{res}\right)\right)$ distribution that the appropriate descriptor of the average value of the variable $\ln\left({\mathrm{D̃}}_{res}\right)$ is simply μ, corresponding to the maximum of the Gaussian distribution $P\left(\ln\left({\mathrm{D̃}}_{res}\right)\right)$ , and also is equal to the median value of the distribution, as $P\left(\ln\left({\mathrm{D̃}}_{res}\right)\right)$ is symmetric.

In the Supporting Information of the original paper (eq S20), we adopted as the average value *D*_*res*_ of the variable ${\mathrm{D̃}}_{res}$ , the arithmetic mean of a log-normal distribution on a linear ${\mathrm{D̃}}_{res}$ scale, namely:

5 $$D_{res}=\exp\left(\mu +\sigma^{2}/2\right)$$

This quantity is mathematically consistent. Different types of average values may be chosen to characterize a distribution. However, the average defined in eq 5 may be strongly biased toward the high- ${\mathrm{D̃}}_{res}$ tail of the distribution when σ is significantly larger than 1, corresponding to distributions extending over more than one decade.

Therefore, the appropriate descriptor is given by the logarithmic average:

6 $$D_{\max}=\exp\left(\mu \right)$$

Accordingly, this definition provides a more representative measure of the central tendency and must be used instead of eq 5 in the main text of the original paper.

In the present case, the standard deviations of the *D*_*res*_ distributions remain relatively small and of comparable magnitude for all samples. Therefore, the use of eq 5 instead of the arithmetic mean does not affect the *relative variations* of the average *D*_*res*_ values across the sets of samples, which are discussed in the paper. This does not change the results and conclusions of the paper, as illustrated in Table 1.

**Table 1:** Values of Residual Dipolar Coupling Constant *D*_res_ and the Fitting Parameters σ and μ of Eq 2, for the Different Subsets of Sulfur Vulcanized HNBR Samples and the Corresponding Nonvulcanized Samples

HNBR	Samples	ACN (wt %)	iRDB (mol %)	μ (=ln 2π *D*_max_)	σ dimensionless	*D*_res_ Calculated with eq 5 (kHz)^a^	*D*_max_ Calculated with eq 6 (kHz)^a^
Therban 3446	HNBR34-NV	34	4	0.95	0.84	0.59	0.41
	HNBR34-0.38			1.16	0.87	0.74	0.51
	HNBR34-0.75			1.35	0.86	0.88	0.61
	HNBR34-1.1			1.42	0.89	0.98	0.66
	HNBR34-1.5			1.42	0.94	1.02	0.66
	HNBR34-1.9			1.44	0.91	1.01	0.67
	HNBR34-2.3			1.59	0.89	1.16	0.78
	HNBR34-3.0			1.58	1.03	1.32	0.77
Therban 3627	HNBR36-NV	36	2	0.99	0.86	0.62	0.43
	HNBR36-0.38			1.21	0.82	0.75	0.53
	HNBR36-0.75			1.34	0.86	0.87	0.61
	HNBR36-1.1			1.42	0.90	0.98	0.66
	HNBR36-1.5			1.50	0.88	1.05	0.71
	HNBR36-1.9			1.50	0.84	1.02	0.72
	HNBR36-2.3			1.51	0.92	1.09	0.72
	HNBR36-3.0			1.50	0.94	1.11	0.71
Therban 4367	HNBR43-NV	43	5.5	1.00	0.88	0.64	0.43
	HNBR43-0.75			1.27	0.90	0.84	0.56
	HNBR43-1.1			1.33	0.90	0.90	0.60
	HNBR43-1.5			1.32	0.97	0.96	0.60
	HNBR43-2.3			1.42	1.07	1.17	0.66
	HNBR43-3.0			1.51	1.07	1.28	0.72
Therban 4498VP	HNBR44-NV	44	9	1.01	0.80	0.60	0.44
	HNBR44-0.38			1.12	0.94	0.76	0.49
	HNBR44-0.75			1.26	0.90	0.84	0.56
	HNBR44-1.1			1.33	0.92	0.92	0.60
	HNBR44-1.5			1.38	0.95	0.99	0.63
	HNBR44-1.9			1.38	0.98	1.02	0.63
	HNBR44-2.3			1.46	1.00	1.13	0.69
	HNBR44-3.0			1.48	1.05	1.21	0.70

a The values of *D*_res_ and *D*_max_ calculated with eqs 5 and 5, respectively, were divided by 2π.

Note, in addition, that the distributions $P\left({\mathrm{D̃}}_{res}\right)$ (with ${\mathrm{D̃}}_{res}$ put in log scale), distinct from the distributions P(ũ) ), are shown in Figure S3.

The corrected version of Figure 4B is shown below. It should also be noted that, based on the above considerations, the quantity σ/D_res_ in the original Figure 4B is not dimensionless, contrary to what was implicitly suggested.

**Figure 4:**
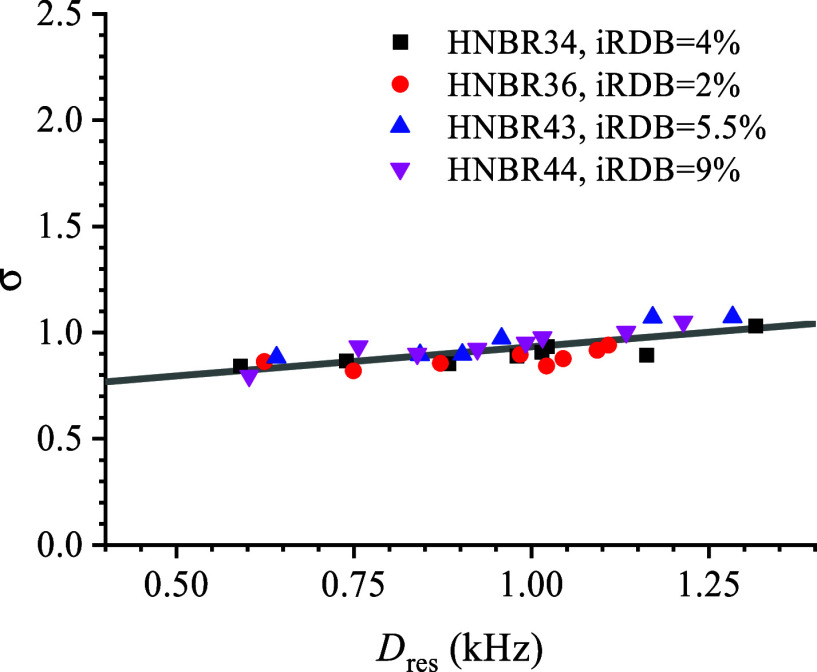
Values of standard deviation of D̃_res_ distribution σ and the average value of D̃_res_ as a function of *D*_res_.

This correction does not affect the conclusions of the paper.

The authors thank Prof. Kay Saalwächter for his valuable suggestion.
